# Context matters: Interpreting effect sizes in education meaningfully

**DOI:** 10.1016/j.mex.2024.103023

**Published:** 2024-10-28

**Authors:** Samuel Tobler

**Affiliations:** ETH Zurich, Zurich, Switzerland

**Keywords:** Effect size, Educational research, Learning sciences, Contextual inferences, Evaluation, Contextual Effect Size Interpretation

## Abstract

Assessing group differences through effect size estimations has become standard practice in educational research. However, the interpretation of these effect sizes is often discussed without considering contextual factors such as prior knowledge, motivation, and socio-economic background that influence the learning outcome. In this methods paper, we discuss the significance of considering affective, cognitive, and sociographic factors when interpreting effect sizes. Furthermore, we propose a theoretical framework for two primary types of effect size distribution based on prior knowledge. This framework may enable researchers in the learning sciences to interpret and contextualize their findings beyond testing null hypotheses and reporting abstracted effect sizes.

- Contextual factors, including prior knowledge or motivation, should be taken into account when interpreting effect sizes.
- In relationship to prior knowledge, effect sizes often follow a normal distribution or an extension thereof following one period of a sine-like wave.

## Graphical abstract

**Figure:**
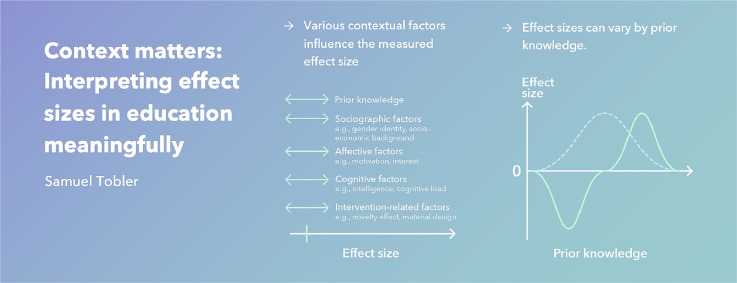

Specifications table

**Table:** 

Subject area:	Psychology
More specific subject area:	Educational Psychology
Name of your method:	Contextual Effect Size Interpretation
Name and reference of original method:	Not applicable.
Resource availability:	Not applicable.

## Background

Recent trends in experimental studies in educational research suggest a move away from simple null hypothesis testing towards supplementing or replacing it with effect sizes or Bayesian analyses [1]. Bayesian analyses assess the probability of observing a difference between groups, while effect sizes measure the magnitude of the difference. In essence, comparing two very similar groups with a slight difference may yield a significant difference if the sample size is large but may not show a significant difference if the sample size is small. Therefore, considering not only the significance but also the effect size can provide more insightful information about potential differences [2].

Meanwhile, the difficulty in interpreting and biases of reporting effect sizes is generally acknowledged [3], and an increasing number of studies report effect sizes in a contextualized manner (cf. [4]). According to Kraft, effect sizes from causal studies in education should be interpreted considering the intervention's cost-effectiveness ratio and scalability. However, it is essential to note that effect sizes only indicate whether there is a difference between the two groups. That it may not fully reveal all underlying aspects of a difference between two groups is evident due to study group-intrinsic and participant-specific characteristics that may vary considerably. Groups can differ based on factors such as prior knowledge, the study setting (including the study administrator, such as a teacher or an external person), the location of the study (whether in a classroom or a laboratory), as well as various cognitive and affective factors including motivation, engagement, or interest. Given these factors, certain differences that may arise can be apprehended through correlation analyses, which help to explore similar trends in two datasets, or mediator analyses, which aim to investigate underlying causal effects that may impact the observed performance difference [5]. However, an individual study only represents a single snapshot of the multidimensional construct researchers are attempting to measure.

To address this limitation, meta-analyses compile individual research findings on a specific topic to identify underlying trends and determine the overall effect size for a particular group comparison [6]. Additionally, meta-analyses often examine moderating factors, which are characteristics that may have influenced the outcome of the results. Moderator analyses aim to categorize the groups of interest based on underlying differences in the study cohorts, such as the grade level at which the study was conducted or differences in experimental design and control conditions. As a result, meta-analyses not only provide the main effect but also identify conditions under which a particular intervention may be more effective (e.g., [7]).

The results of meta-analytical studies, along with information resulting from moderator variables, are extremely valuable for educators, curriculum developers, and researchers who want to understand the current state of research. However, in the context of individual studies in educational research, the implications of obtained results from specific group comparisons may not be immediately clear. In this opinion piece, we emphasize the importance of considering the contextual impact of cognitive and affective factors when examining effect size. We propose a way to understand effect sizes and introduce two types of effect size distributions and their presence in educational studies.

## Method details

### *Effect sizes are influenced by prior knowledge and cognitive and affective factors*

In educational settings, the ability to learn something new depends on various factors, including motivation, interest, intelligence, and, most importantly, prior knowledge. Connecting new information to understood concepts and integrating new ideas into existing knowledge frameworks is crucial for learning [8]. Therefore, students' individual topic-specific prior knowledge, combined with their out-of-school experiences, plays a crucial role in any learning situation and should be considered when designing educational interventions. Moreover, different instructional interventions might promote different student cohorts. However, even though the aim of a specific instruction might differ for distinct scenarios (e.g., promoting gifted students vs. closing the performance gap in general education), the effectiveness of an intervention depends on prior knowledge.

Next to this essential interdependence of prior knowledge and post-test outcome, various student-specific and context-dependent factors come into play that may influence the ultimately observable effect size. These factors include students' (gender) identity, socio-economic background, cultural influences, first-generation college status, access to educational resources, and experiences of discrimination or bias, all of which can influence students' behavior in the classroom (e.g., [[9], [10]]).

In addition to sociographic factors, the design of educational interventions is crucial. The use of new technologies, such as VR- or AR-grounded instructions or exercises involving AI-based tools, may enhance students’ engagement due to the novelty of these tools in the classroom (e.g., [11]). Similarly, motivating students, increasing their interest in learning something new, or affecting their perceived satisfaction with an experimental condition influences their learning behavior and, thus, performance (e.g., [12]). From a cognitive perspective, reducing components that increase extraneous cognitive load or implementing others that increase germane cognitive load may impact students' learning [13]. Furthermore, individual differences in psychometric intelligence also affect the ability and efficiency of information processing [14].

**Fig. 1:**
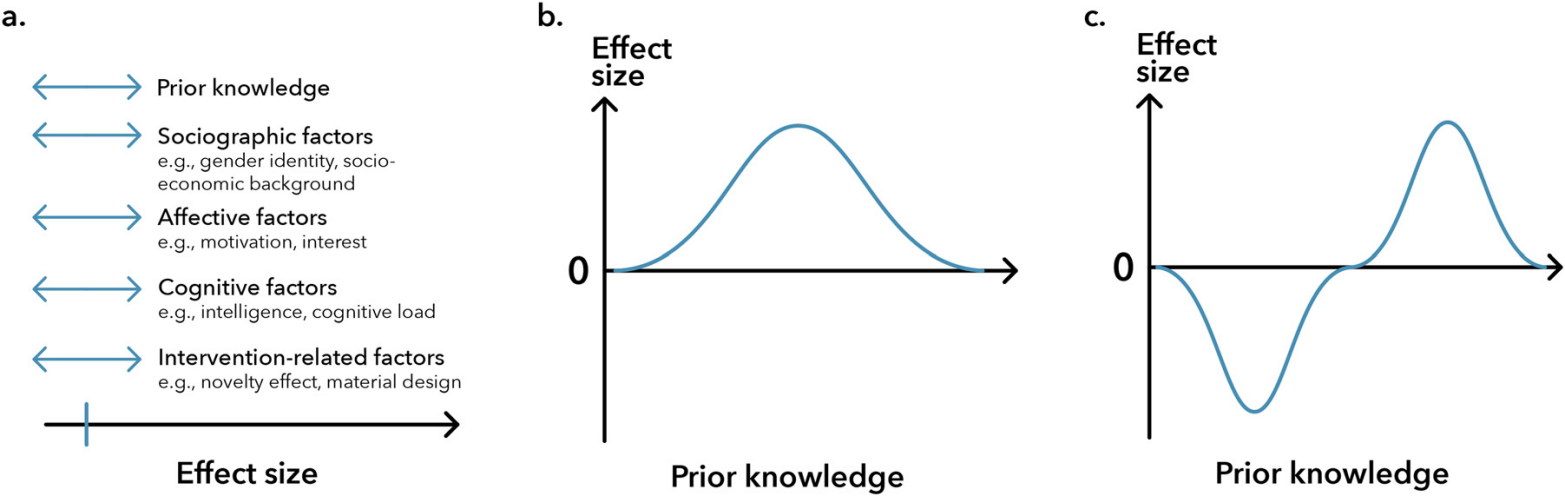
Influence of moderating factors on effect size (a) and effect size-performance dependencies (b-c).

The more factors that influence the outcome of an intervention, the greater the individual differences in a group of students. Consequently, the distribution of post-test performance becomes more varied as these factors affect each student differently, which reduces the likelihood of finding significant differences between groups. Therefore, it is crucial to move away from simple significance testing and consider the effect size in combination with the factors influencing its magnitude (Fig. 1a). To address this, interventions and control conditions should be designed with minimal variable changes, experiments could be conducted in a controlled laboratory setting, and specific questionnaires could be used to assess these factors. However, it remains unlikely that all influencing variables can be excluded in an educational study because the assessment of these variables is often limited and subject to biases. Therefore, greater consideration should be given to underlying factors and contextual elements when discussing experimental findings.

### *The two main types of expectable effect size distributions*

Acknowledging that various factors influence the prior knowledge-dependent post-intervention performance outcome in educational studies, we suggest that the measured difference in effect sizes follows two main distribution types, which we will discuss next. Both of these types share a common starting and ending point. When students have insufficient prior knowledge to understand the educational material, the effect size difference after the instruction phase is zero, indicating no learning gains or performance differences. Similarly, if students fully understand the covered topic, their performance outcome will also be zero. Thus, finding an effect size that is unequal to zero indicates that the obtained results lie somewhere between these two maxima.

The first type of effect size distribution describes a situation where the effect size between the two maximum values follows a normal distribution (Fig. 1b). If students have prior knowledge, they may be able to build on it and integrate new information into their existing knowledge framework. Similarly, the better their prior knowledge aligns with the instructional needs, the greater the measured effect size difference will be. As students' existing knowledge increases, the observable effect size will decrease until it reaches zero.

The second type of distribution is an extension of the normal distribution, reflected across the x-axis, resembling one period of a sine-like wave (Fig. 1c). An intervention may benefit students with lower prior knowledge but could lead to lower performance for those with higher prior knowledge. In other words, the same educational intervention could have zero, positive, or even negative effects. The underlying reason for this pattern might be explained by contextual factors. Certain aspects of the intervention may have been beneficial for students with less prior knowledge but distracting for those with more prior knowledge, or the educational materials may have been too challenging for students with less prior knowledge while those with more prior knowledge were appropriately challenged and benefited more from the intervention. The potential reasons for such differences in effect size are numerous and must be analyzed on a case-by-case basis.

Hence, the effect size reported in experimental studies in educational research ultimately depends on various cognitive and affective factors that influence the magnitude and are likely to be found in one of these two distributions.

## Concluding remarks

When conducting research in educational settings, it is important to critically reflect on the different factors that can influence the measured effect size and its potential distribution. Considering which confounding factors could affect the outcome before any classroom intervention might help counter specific aspects by the intervention design or by developing questionnaires to quantitatively investigate the impact of these aspects at the intervention time point. Consequently, the subsequent discussion of the effect size difference upon having conducted the study and analyzed the results can be performed contextually. Moreover, null results or findings that are contrary to the general trend of research in a specific field may be more easily explained. Therefore, comprehending the dimensions of an effect size and being able to assess the underlying factors that may impact the extent of group differences can not only aid in adjusting research designs and identifying relevant confounding variables but also in generating new research questions. Eventually, it can also provide valuable insights to educators when developing learning materials for their classrooms and to curriculum developers and educational reformists when crafting policies and strategies for educational advancement.

## Limitations

Not applicable.

## Ethics statement

Not applicable.

## Credit author statement

ST is the only contributor to this work.

## Declaration of competing interest

The authors declare that they have no known competing financial interests or personal relationships that could have appeared to influence the work reported in this paper.
